# Flexor and Extensor Ankle Afferents Broadly Innervate Locomotor Spinal Shox2 Neurons and Induce Similar Effects in Neonatal Mice

**DOI:** 10.3389/fncel.2019.00452

**Published:** 2019-10-09

**Authors:** Erik Z. Li, D. Leonardo Garcia-Ramirez, Kimberly J. Dougherty

**Affiliations:** Department of Neurobiology and Anatomy, Drexel University College of Medicine, Philadelphia, PA, United States

**Keywords:** locomotion, central pattern generator, spinal cord, interneuron, sensory input

## Abstract

Central pattern generators (CPGs) in the thoracolumbar spinal cord generate the basic hindlimb locomotor pattern. The locomotor CPG integrates descending commands and sensory information from the periphery to activate, modulate and halt the rhythmic program. General CPG function and response to sensory perturbations are well described in cat and rat models. In mouse, roles for many genetically identified spinal interneurons have been inferred from locomotor alterations following population deletion or modulation. However, the organization of afferent input to specific genetically identified populations of spinal CPG interneurons in mouse remains comparatively less resolved. Here, we focused on a population of CPG neurons marked by the transcription factor Shox2. To directly test integration of afferent signaling by Shox2 neurons, sensory afferents were stimulated during patch clamp recordings of Shox2 neurons in isolated spinal cord preparations from neonatal mice. Shox2 neurons broadly displayed afferent-evoked currents at multiple segmental levels, particularly from caudal dorsal roots innervating distal hindlimb joints. As dorsal root stimulation may activate both flexor- and extensor-related afferents, preparations preserving peripheral nerves were used to provide more specific activation of ankle afferents. We found that both flexor- and extensor-related afferent stimulation were likely to evoke similar currents in a given Shox2 neuron, as assessed by response polarity, latency, duration and amplitude. It has been proposed that Shox2 neurons can be divided into neurons which contribute to rhythm generation and neurons that are premotor by the absence and presence of the V2a marker Chx10, respectively. Response to afferent stimulation did not differ based on Chx10 expression. Although currents evoked in response to flexor and extensor afferent activation did not follow expected functional antagonism, they were consistent with the observation that stimulation of flexor- and extensor-related afferents both reset the phase of ongoing fictive locomotion to flexion in neonatal mice. Together, the data suggest that Shox2 neurons are interposed in multiple sensory pathways and low threshold proprioceptive input reinforces sensory perturbation of ongoing locomotion by similarly activating or inhibiting both the rhythm and patterning layers of the CPG.

## Introduction

Locomotion is an essential part of the behavioral repertoire of many animals for seeking resources or escaping danger and is frequently accomplished by means of a repetitive motor pattern. In humans and other vertebrates, neural circuits in the spinal cord known as locomotor central pattern generators (CPGs) are capable of generating this basic motor pattern. In humans, these circuits are localized in the thoracolumbar spinal cord and an analogous region exists for hindlimb locomotion in quadrupedal animals such as cats and rodents. Although CPG circuits can independently generate the basic rhythm and alternating motor pattern, descending control from supraspinal structures and ascending sensory inputs from proprioceptors and cutaneous afferents are normally integrated to enable skilled and automated movements (Goulding, 2009; Kiehn, 2016). Understanding CPG circuit organization and sensory modulation of the circuit may be essential to develop strategies for locomotor recovery in several pathologies including spinal cord injury, stroke and multiple sclerosis.

Sensory afferents play an important role in the generation and modulation of context-appropriate shifts in locomotor stance/swing timing and muscle force. Experiments in cats and rats have allowed very fine partitioning of sensorimotor function during locomotion and resulted in significant advances in understanding CPG control (McCrea, 2001; Rossignol et al., 2006). For example, spinally transected cats can adapt locomotor frequency to match treadmill speed and this behavior is abolished following dorsal root transection (Andersson et al., 1978; Grillner and Rossignol, 1978). In a similar fashion, sinusoidal movements of the hip can entrain ongoing locomotor-like nerve outputs in an animal model treated with a neuromuscular blocker (Andersson and Grillner, 1983). Throughout the stance phase, input from ankle extensors also provide speed-dependent enhancement of force generation in hindlimb extensor muscles (Duysens and Pearson, 1980; Conway et al., 1987; Gossard et al., 1994; Guertin et al., 1995; Mayer et al., 2018).

In addition to relatively long-lasting sensory inputs, locomotor generation is also sensitive to acute sensory signals. Brief electrical stimulation of ankle extensors in the cat terminates ongoing flexor activity and initiates extensor activity (Conway et al., 1987). In the normal transition of stance to swing, hip angle at swing onset is relatively constant across a range of locomotor tasks (Lam and Pearson, 2001; McVea et al., 2005). Furthermore, acute stretch of hindlimb flexor muscles at the hip and ankle can also initiate a new flexor phase (Perreault et al., 1995; Hiebert et al., 1996; Stecina et al., 2005). Additionally, activation of the foot cutaneous afferents evokes a stumbling corrective response to avoid an obstacle (Prochazka et al., 1978; Wand et al., 1980; Mayer and Akay, 2018).

The ability of sensory signals to entrain and perturb locomotion strongly implies that sensory afferents have access to CPG circuit elements, likely through multiple distinct and possibly overlapping pathways. In the cat and rat models, these effects are well established, but it has been difficult to determine the CPG neuronal elements which receive and integrate sensory information, partly because no clear anatomic nuclei can reliably identify such interneurons. Similarly, computational models based on the cat and rat experimental results are limited in the data available to directly identify classes of spinal neurons comprising the CPG (Brown, 1911; Pearson and Duysens, 1976; Grillner, 1981; McCrea, 2001; Rybak et al., 2006a, b; Guertin, 2009). Sensory modulation of locomotor activity is also known to occur in the mouse, but the effects are less fully described (Hinckley et al., 2010; Akay et al., 2014; Takeoka et al., 2014). However, by leveraging transgenic tools available in the mouse model, many neuronal populations with locomotor functions have been identified and a putative circuit architecture has been proposed (for review see Rybak et al., 2015). Data from the cat and rat and from the mouse are therefore complementary in that sensory effects are well-described in the cat and rat but circuit elements are difficult to identify, and vice-versa in the mouse. Therefore, understanding the mechanisms by which sensory information is conveyed to CPG neurons in the mouse will not only provide novel information regarding CPG circuit organization and connectivity, but will also provide an important link between these two bodies of literature.

Sensory perturbations which have strong locomotor effects are likely to be mediated by neurons which participate in the generation of locomotor rhythm. Although a marker which can reliably and specifically label all such neurons remains elusive, specific genetically identified populations of neurons are thought to comprise subsets of the rhythm-generating (RG) kernel (Brownstone and Wilson, 2008; Dougherty and Ha, 2019). Here, we focus on neurons identified by the transcription factor Shox2, a subset of which have been proposed to play a role in locomotor rhythm generation (Dougherty et al., 2013).

The goals of this study were to determine how proprioceptive afferent information reaches Shox2 neurons and affects fictive locomotion in mouse. We demonstrate that Shox2 neurons receive broad innervation from sensory afferents in the quiescent state, likely mediated by a minimally disynaptic pathway. Based on effects seen in the cat and rat, we hypothesized that low-threshold input from ankle extensor and flexor afferents would induce opposing effects in Shox2 neurons. Instead, we found that most Shox2 neurons receive similar postsynaptic currents following both ankle extensor stimulation and ankle flexor stimulation which were predominantly inhibitory and could be long duration. Shox2 neurons are known to overlap with the V2a population and more specific labeling of RG neurons can be accomplished by specifically targeting non-V2a Shox2 neurons (Shox2^RG^). The V2a Shox2 subpopulation (Shox2^PF^) is thought to be enriched for downstream interneurons which recruit motor neurons, a function that has been referred to as pattern forming (PF) (Rybak et al., 2006a, b, 2015). Shox2^PF^ neurons which responded to ankle afferent stimulation were preferentially situated in rostral lumbar segments, but the response patterns otherwise did not differ between Shox2^RG^ and Shox2^PF^ neurons. This corresponded with the finding that both ankle flexor and extensor afferent stimulation reset ongoing fictive locomotion by activating flexor motor pools. Taken together, this suggests that low threshold proprioceptive input may reach rhythm and patterning layers similarly, thereby reinforcing the sensory effect on locomotion at both levels.

## Materials and Methods

All experimental procedures were approved by the Institutional Animal Care and Use Committee at Drexel University and followed the guidelines of the National Institutes of Health for laboratory animal welfare. Experiments were performed using Shox2::Cre (Dougherty et al., 2013); Rosa26-flox-Stop-flox-tdTomato (Ai9 from Jax Mice, #007909, Madisen et al., 2010) or in Shox2::Cre;Ai9;Chx10eGFP (also called Vsx2-eGFP, MMRRC, 011391-UCD, Gong et al., 2003) transgenic mice.

### Spinal Cord Preparations

Spinal cords preparations were isolated from postnatal day (P)1 to P4 mice. Briefly, mice were decapitated and eviscerated, after which the vertebral bodies were removed to expose the spinal cord. The spinal cord and attached dorsal root ganglia (DRG) were then removed in ice cold dissecting solution. Spinal cord preparations were isolated in a dissecting solution bubbled with 95% O_2_/5% CO_2_ (carbogen) containing in mM: 111 NaCl, 3 KCl, 11 glucose, 25 NaHCO_3_, 3.7 MgSO_4_, 1.1 KH2PO_4_, and 0.25 CaCl_2_.

#### Peripheral Nerve Dissection

In some experiments, the dissection was extended to include hindlimb peripheral nerves. Care was taken during removal of the vertebral bodies to avoid damaging spinal nerves. Hindlimb muscles were removed to expose the sciatic nerve and its branches, which were then dissected free. In mice, there is strain-specific variation in lumbar spinal cord anatomy (Rigaud et al., 2008). Spinal roots from the third and fourth lumbar levels are the primary contributions to the sciatic nerve in the mice used for this study.

#### Accessing Shox2 Neurons

In order to visualize Shox2 neurons for whole cell patch clamp, it was necessary to create a tissue window. To prevent compression of the cord, spinal meninges were first carefully removed or split over incision sites. In experiments using a ventral horn-removed preparation, a section of ventral horn was removed (L2-S2) using a surgical microknife (5.0 mm cutting edge, 15.0 cutting angle, Fine Science Tools #10315-12) to gain visual access to spinal interneurons for patch clamp. For experiments using a hemisect preparation, a unilateral section of spinal cord (L2-S2) was removed. Following isolation, spinal cords were transferred to room temperature (RT) artificial cerebral spinal fluid (ACSF) recording solution containing in mM: 111 NaCl, 3 KCl, 11 glucose, 25 NaHCO_3_, 1.3 MgSO_4_, 1.1 KH_2_PO_4_, and 2.5 CaCl_2_. Cords were incubated in carbogen-bubbled ACSF for a minimum of 30 min prior to recording.

#### Preparations From Older Mice

In subsets of some experiments, mice aged P5–P14 were used. Mice <P8 were dissected using the procedures described above. Mice P8 and older were first anesthetized with isoflurane prior to decapitation and spinal cords were isolated using an alternative glycerol-based dissecting solution bubbled with carbogen and containing in mM: 3 KCl, 11 glucose, 25 NaHCO_3_, 1.3 MgSO_4_, 1.1 KH_2_PO_4_, 2.5 CaCl_2_, 222 glycerol. Following isolation, these cords were incubated in carbogen-bubbled recording ACSF at 37°C for 30 min and then allowed to equilibrate to RT for 30 min prior to recording.

### Electrophysiology

#### Patch Clamp

Shox2 neurons were recorded in ACSF and visually identified at 63× on an Olympus BX51WI microscope with LED illumination (X-Cite) by red fluorescence (Semrock Brightline CY3-4040C). In cords from Shox2::cre;Ai9;Chx10eGFP mice, Shox2 neurons were further specified by the V2a-specific marker Chx10 into the Chx10-negative Shox2^RG^ or the Chx10-positive Shox2^PF^ based on green fluorescence (Semrock Brightline FITC-3540C). Intracellular electrodes were pulled to 5–8 MΩ using a Sutter P-1000 and filled with intracellular solution containing in mM: 128 K-gluconate, 10 HEPES, 0.0001 CaCl_2_, 1 glucose, 4 NaCl, 5 ATP, and 0.3 GTP. Cells were recorded in whole-cell configuration from the soma using a Multiclamp 700B and digitized at 10–20 kHz with an Axon Digidata 1550A connected to a PC tower running Clampex.

#### Afferent Stimulation and Spinal Root Recordings

During recordings of Shox2 neurons, dorsal roots or peripheral nerves were stimulated with tight-fitting glass suction electrodes to activate afferent sensory pathways. Ventral roots were cut free proximal to the spinal nerve to prevent transmission of antidromic axon potentials in efferent motor pathways. Dorsal root and peripheral nerves were stimulated using 50 μs square pulses with a 10 s interstimulus interval to reduce effects of short-term synaptic plasticity. Pulse waveforms were generated by either an Axon Digidata 1550A or an AMPI Master-9 pulse stimulator connected to an optical stimulus isolator. In some experiments, afferent volleys in dorsal roots or ventral root reflexes were also recorded. Signals were amplified 1000× and bandpass filtered from 10 Hz to 1 kHz using a model MA 102 amplifier (custom built in the workshop of the Zoological Institute, University of Cologne, Germany), then digitized at 100 kHz with an Axon Digidata 1550A connected to a PC. Where reported, stimulus threshold (1 × T) for afferent volley or ventral root reflex was defined as the minimum current respectively necessary to reliably evoke dorsal root or ventral root responses in 10/10 trials.

#### Postsynaptic Current Analysis

Post-stimulation currents recorded in Shox2 neurons were categorized as stimulation-evoked if the response was present in at least 8 out of 10 stimulation trials. Current components were identified as inhibitory or excitatory based on reversal potential (inhibitory < −35 mV, excitatory > −5 mV). Current properties including latency, peak amplitude and duration were measured using Clampfit 10 (Molecular Devices). Latency was measured as the difference between the time that the current exceeded pre-stimulus baseline levels and the time of stimulation. Jitter was defined as the standard deviation in the latency of the earliest current components. Amplitude is reported and analyzed as absolute value. Where data is presented using box and whisker plots, the boxplot labels median, 1st quartile and 3rd quartile, with whiskers extending to 1.5× the interquartile range beyond the 1st and 3rd quartiles.

#### Measurement of Cell Location

Many genetically labeled interneuron populations show differences in function or connectivity that is dependent on cellular position. In this study, cellular position was measured and reported as segmental level, mediolateral position and dorsoventral position. Distances were measured from images captured at 10× immediately following cell recordings and before removing the patch electrode. These measurements were then normalized according to the following procedures. The center point of the dorsal root at the entry zone is designated as segment number and Shox2 segmental level is normalized such that the distance between each dorsal root entry zone center point is 1. Mediolateral position is reported as the distance between the cell and the midsagittal plane divided by distance between the lateral tissue border and the midsagittal plane, resulting in a normalized value from 0 (medial) to 1 (lateral). Similarly, dorsoventral position is reported as the distance between the cell and the dorsal tissue border divided by the distance between the dorsal and ventral tissue borders, resulting in a normalized value from 0 (dorsal) to 1 (ventral).

#### Locomotor Experiments

Spinal cords were prepared from P1–P3 mice with attached peripheral nerves. Fictive locomotion was evoked with 7 μM *N*-methyl-D-aspartate (NMDA) and 8 μM serotonin (5-HT). Cords were allowed to equilibrate with the locomotor cocktail for at least 15 min prior to recording. Flexor- and extensor-related locomotor activity were respectively recorded from L1 and L4 ventral roots using tight-fitting glass suction electrodes and digitized at 10 kHz. Locomotor burst envelopes were generated by rectifying and low-pass filtering at 0.25–0.55 Hz. Locally-weighted scatterplot smoothing (LOWESS) with a 3–60 s window was used to generate a locally adaptive signal threshold for burst onset and offset. For perturbation experiments, trains of electrical stimulation (4–6 pulses, 50 μs pulse duration, 20 Hz) were delivered to peripheral nerves in 100 s intervals. In these preparations, ventral roots were transected proximal to the spinal nerve to prevent antidromic transmission along motor fibers.

#### Statistical Tests and Reporting

Univariate descriptive statistics were reported as mean ± standard deviation and range unless otherwise specified. Statistical tests were performed using the R 3.6.0 software package with a criterion α < 0.05 for significance. All statistical tests were performed on raw values even when plotted on log-axes for visualization. Categorical data was analyzed using Chi-squared tests, with Yate’s continuity correction used for 2 × 2 contingency tables. For comparisons between groups of continuous variables, normality was first tested using the Shapiro–Wilk test and homogeneity of variances (homoscedasticity) was tested using the Fligner-Killeen test of homogeneity of variances. For two groups, comparisons of normally-distributed data were performed using Welch’s unequal variance *t*-tests. For more than two groups, comparisons of normally-distributed data were performed with one-way ANOVA for homoscedastic data and Welch’s unequal variances ANOVA for heteroscedastic data. *Post hoc* comparisons following non-parametric ANOVAs were performed with Tukey’s honest significant differences test. Comparisons of non-normal data were performed with the Wilcoxon rank sum test (Mann–Whitney *U* Test) for two groups or Kruskal–Wallis rank sum test (parametric ANOVA) for more than two groups. When applied to heteroscedastic data, parametric tests were interpreted as tests of stochastic dominance rather than location. *Post hoc* comparisons following Kruskal–Wallis rank sum tests were performed using Dunn’s test corrected for multiple comparisons using the Holm–Bonferroni method (Dinno, 2015).

## Results

### Shox2 Neurons Receive Input From Sensory Afferent Pathways

**FIGURE 1:**
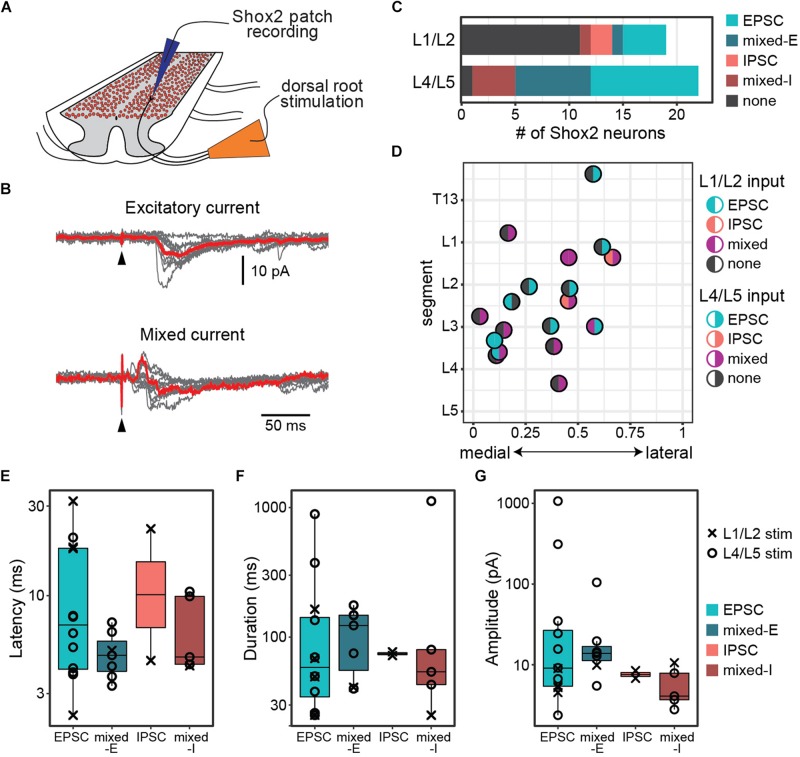
Postsynaptic currents are detected in Shox2 neurons following dorsal root stimulation. **(A)** Cartoon of experimental setup for the ventral horn-removed experiments. Dorsal roots were stimulated while recording from Shox2 neurons (red) in lumbar segments of cords isolated from Shox2::cre;Rosa26-lsl-tdTomato mice. **(B)** Postsynaptic currents were recorded in Shox2 neurons in response to stimulation of rostral (L1 or L2) and/or caudal (L4 or L5) lumbar dorsal roots. Evoked currents in Shox2 neurons were only excitatory (EPSC), only inhibitory (IPSC), or mixed with shortest latency components that were excitatory (mixed-E) or inhibitory (mixed-I). Top: example showing excitatory current. Gray traces show 10 individual trials and red trace shows average. Black triangle indicates stimulation artifact. Bottom: example showing mixed-I current. **(C)** Number of recorded Shox2 neurons displaying postsynaptic currents in response to stimulation of dorsal L1/L2 (8/19) and L4/L5 roots (21/22). **(D)** Responses of Shox2 neurons in which both L1/L2 and L4/L5 stimulation were tested are plotted according to lumbar level and mediolateral position. The color of each point is based on response (EPSC in teal, IPSC in pink, mixed in purple, and gray for no response) following L1/L2 (left half) and L4/L5 (right half) stimulation. The center point of the dorsal root at the entry zone is designated as segment number. **(E–G)** Latency, duration, and amplitude were characterized for each response to L1/L2 (x) and L4/L5 (o) stimulation and plotted on log scale for better visualization of points.

Sensory inputs may have either local or distant effects on spinal circuits. Ankle afferents, in particular, have been shown to influence activity at hip and knee joints (Duysens and Pearson, 1980; Angel et al., 1996). We therefore first investigated the distribution of Shox2 neurons receiving sensory input from different segmental regions by determining whether Shox2 neurons received postsynaptic currents in the quiescent state following afferent stimulation at rostral and caudal lumbar levels. Shox2 neurons are located in the intermediate and ventral regions of the spinal cord which is fortuitous for various reduced preparations to visually access these neurons for whole cell patch clamp recordings. For these experiments, the most ventral part of the spinal cord was removed from isolated spinal cords prepared from P1–P14 mice, allowing fluorescently labeled Shox2 neurons to be visualized for whole-cell patch clamp recordings (Figure 1A). Rostral (L1 or L2) and caudal lumbar (L4 or L5) dorsal roots were stimulated while Shox2 neurons were monitored with whole cell recordings.

Recorded postsynaptic currents were considered to be stimulation evoked if they were present in at least 8 out of 10 trials (Figure 1B). Nearly all Shox2 neurons displayed postsynaptic currents in response to L4/L5 DR stimulation (95%, 21/22 from 12 mice). Currents were also observed in Shox2 neurons following L1/L2 DR stimulation, albeit less often (42%, 8/19 from 9 mice; Figure 1C). Threshold for L4/L5-evoked response in Shox2 neurons (39.81 ± 22.60 μA, range 15.00–100.00 μA) was lower than for L1/L2-evoked responses (124.38 ± 93.33 μA, range 60.00–350.0 μA) (Wilcoxon rank sum test, *W* = 160.5, *p* = 0.0002, data not shown). Evoked currents recorded in Shox2 neurons often had more than one component and could be divided into those containing purely excitatory components, purely inhibitory components, or both (mixed responses). Mixed responses could be further divided into those with shortest latency components that were excitatory (mixed-E) or inhibitory (mixed-I). L1/L2 stimulation elicited a mix of all response types in the Shox2 neurons with observed evoked currents. Shox2 neurons displayed EPSCs or mixed currents following L4/5 stimulation, but not currents composed solely of IPSCs. However, when considering only Shox2 neurons in which a postsynaptic current was observed, no difference was detected in the distribution of these response subtypes when comparing L1/L2 and L4/L5 dorsal root stimulation (χ^2^ = 6.31, df = 3, *p* = 0.10). In a subset of Shox2 neurons (18/23), the response to both L1/L2 and L4/L5 DR stimulation was tested (Figure 1D). No association was observed between current presence/absence following L1/L2 and following L4/L5 DR stimulation (χ^2^ = 0.055, df = 1, *p* = 0.81). Currents evoked in Shox2 neurons following L1/L2 and L4/L5 dorsal root stimulation had similar proportions of current response types (χ^2^ = 6.31, df = 3, *p* = 0.097). We were also unable to detect any segmental variation in Shox2 neuron response presence for L1/L2 stimulation (Kolmogorov–Smirnov test, segmental level of neurons receiving or not receiving currents following L1/L2 stimulation: *D* = 0.18, *p* = 0.98) or L4/L5 stimulation (all but 1 tested neuron responded to L4/L5 stimulation).

To better understand the effects of afferent stimulation on Shox2 neurons, we characterized the response latency, duration and amplitude (Figures 1E–G). Most (85.7%, 18/21) Shox2 neurons which responded to L4/L5 stimulation responded to currents at strengths of 50 μA, so responses were characterized at this strength to reduce the possibility of activating high-threshold nerve fibers. Neurons responding to L1/L2 typically required much stronger dorsal root stimulation, so currents resulting from L1/L2 stimulation were characterized close to the threshold to observe a current response (<20 pA above threshold). Overall, response latencies were 8.65 ± 7.37 ms (range 2.3–31.9 ms) from the stimulation, suggesting that sensory stimulation can access Shox2 neurons through multiple pathways with differing numbers of interposed interneurons. No difference in latency distribution was observed between responses with an initial excitatory or inhibitory component (Kolmogorov–Smirnov test, *D* = 0.42, *p* = 0.90), nor could we detect a difference in latency between different response types (Kruskal–Wallis rank sum test, χ^2^ = 2.34, df = 3, *p* = 0.505). Since inhibitory and excitatory responses showed no difference in response latency, it is likely that most inputs to Shox2 neurons are not monosynaptic. Response durations (159.0 ± 261.64 ms, range 24.97–1120.0 ms), here considered as the total duration of the multicomponent input, and postsynaptic current peak amplitudes (66.21 ± 213.02 pA, range 2.38–1066.66 pA) were highly variable and similarly showed no difference between different response subtypes (Kruskal–Wallis rank sum test, duration: χ^2^ = 1.01, df = 3, *p* = 0.80; amplitude: χ^2^ = 5.52, df = 3, *p* = 0.14). Further, there were no significant differences between L1/2 and L4/5 evoked EPSCs in Shox2 neurons for any measure described (Wilcoxon rank sum test, latency: *W* = 22, *p* = 0.37; duration: *W* = 13.5, *p* = 0.73; amplitude: *W* = 27, *p* = 0.073). In summary, we observe that Shox2 neurons across the lumbar segmental and mediolateral extent of the cord respond robustly to L4/L5 stimulation, likely through a heterogeneous set of sensory processing pathways.

### Stimulation of Both Common Peroneal and Tibial Nerves Induce Postsynaptic Currents in Shox2 Neurons

In the lumbar region of the spinal cord, ventral root activity from the rostral and caudal levels has been found to correspond respectively to flexor and extensor motor activity in the hindlimb. However, a single ventral or dorsal root innervates multiple muscle groups that can span several joints and may include both flexors and extensors (Vejsada and Hník, 1980; Peyronnard and Charron, 1983). Dorsal root stimulation at any lumbar level is likely to activate both flexor- and extensor-related proprioceptive pathways simultaneously. Therefore, evoked currents measured in Shox2 neurons in response to dorsal root stimulation could result from activation of pathways that are flexor-related, extensor-related or both.

**FIGURE 2:**
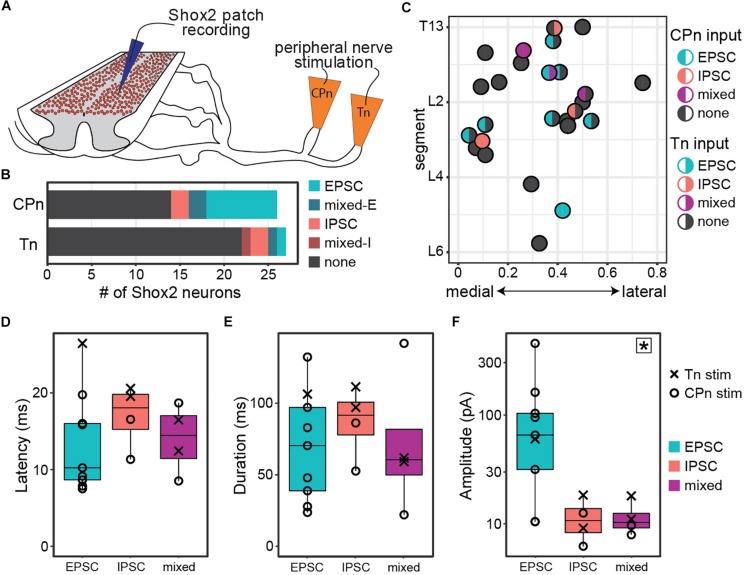
Both common peroneal and tibial nerve stimulation can induce postsynaptic currents in Shox2 neurons. **(A)** Cartoon of ventral horn-removed spinal cord preparation with intact sciatic nerve branches for stimulation of common peroneal (CPn) and tibial nerves (Tn). **(B)** The proportion of excitatory, inhibitory, mixed-E, and mixed-I currents detected in Shox2 neurons following CPn stimulation and following Tn stimulation. **(C)** Shox2 neurons recorded during stimulation of both CPn and Tn are plotted according to anatomical position and color-coded based on response type. Segment number corresponds to the center point of the dorsal root at the entry zone. Shox2 neurons displaying postsynaptic currents to CPn and Tn stimulation were observed throughout the lumbar cord. **(D–F)** Latency, duration and amplitude plotted for each response subtype. Mixed-E and mixed-I responses were pooled into a single mixed subtype. Latency and duration did not significantly differ between evoked excitatory, inhibitory or mixed currents, whereas peak amplitude differed significantly between current types (*p* = 0.027, indicated by star in upper right corner). *Post hoc* testing did not detect significant pairwise comparisons when corrected for multiple comparisons (see text for details). Amplitude plotted on log scale for clarity.

To address this issue, we modified our ventral horn-removed preparation to include preserved hindlimb peripheral nerves and isolated common peroneal (CPn) and tibial nerves (Tn) to provide more specific activation of flexor- and extensor-related proprioceptors around the ankle joint (Figure 2A). For these experiments, neonatal mice ≤ P7 were utilized. The CPn suction electrode was attached at the level of the fibular neck to activate flexor-related afferents. The Tn suction electrode was attached proximal to the nerve branches innervating the medial and lateral gastrocnemius (extensor) muscles. Stimulation threshold for currents evoked in Shox2 neurons was found to be 60.2 ± 48.4 μA for CPn and 70.0 ± 54.3 μA for Tn. Therefore, presence and properties of evoked currents were characterized from 100 to 150 μA stimulation, which was sufficient to evoke reliable responses in most Shox2 neurons receiving input from either nerve.

Postsynaptic currents were detected in 46% (12/26 from 12 mice) of tested Shox2 neurons following CPn stimulation and 18% (5/27 from 13 mice) following Tn stimulation. Currents could be divided into excitatory, inhibitory or mixed responses as described above. The proportion of response types was not significantly different between responses to CPn and Tn stimulation (χ^2^ = 4.69, df = 3, *p* = 0.20; Figure 2B). As only 4 responses were mixed, these were not subdivided into mixed-E and mixed-I populations for further analyses.

In 96% (26/27 from 13 mice) of recorded neurons, both Tn and CPn stimulation were tested (Figure 2C). Presence or absence of currents evoked in response to CPn stimulation was not found to be associated with Tn evoked current presence (χ^2^ = 1.42, df = 1, *p* = 0.23). Shox2 neurons located throughout the lumbar cord responded to both CPn and Tn stimulation (Figure 2C). No differences were detected in the segmental distribution of Shox2 neurons on the basis of CPn stimulation response or Tn stimulation response presence (Kolmogorov–Smirnov test, segmental level of neurons receiving or not receiving currents following CPn stimulation: *D* = 0.20, *p*-value = 0.90; following Tn stimulation: *D* = 0.37, *p* = 0.50). Further, there were no apparent differences in medial-lateral distribution. 15% (4/26) of Shox2 neurons displayed postsynaptic currents in response to both CPn and Tn stimulation. In these neurons, 75% (3/4) of neurons showed concordance in current type following CPn and Tn stimulation, but these included excitatory, inhibitory and mixed current response types (Figure 2C). In the fourth neuron, CPn and Tn stimulation both induced early excitatory postsynaptic currents, but there was also a longer latency inhibitory current (mixed-E) following Tn stimulation.

We also characterized the latency, duration, and amplitude of these evoked currents (Figures 2D–F). Overall latencies were 14.46 ± 5.52 ms (range 7.51–26.47 ms). No significant differences in latency or duration were detected between excitatory, inhibitory or mixed response types (Kruskal–Wallis rank sum test, latency: χ^2^ = 2.29, df = 2, *p* = 0.32; duration: χ^2^ = 1.0392, df = 2, *p* = 0.59). Primary afferents fibers are glutamatergic, so inhibitory postsynaptic currents measured in Shox2 neurons are mediated by minimally disynaptic pathways. As excitatory and inhibitory response latencies are comparable, we therefore suggest that both excitatory and inhibitory responses are primarily mediated by minimally disynaptic pathways. However, we observed a subset of cells with excitatory currents (5/9) with latencies shorter than the earliest inhibitory responses observed (Figure 2D, <11.34 ms); thus we cannot rule out the possibility of monosynaptic excitatory connections between primary afferents and Shox2 neurons. Current amplitudes differed between excitatory, inhibitory and mixed currents, but *post hoc* testing was unable to identify which currents differed in pairwise comparisons when corrected for multiple comparisons (Kruskal–Wallis rank sum test, χ^2^ = 7.26, df = 2, *p* = 0.027; *post hoc* Dunn’s test, EPSC-IPSC: *p* = 0.088, EPSC-Mixed: *p* = 0.059, IPSC-Mixed: *p* = 1.0). Larger EPSC amplitudes may be expected, given that both resting membrane potential and the holding potential for measurements (−50 mV) are near the chloride reversal potential. Taken together, only subsets of Shox2 neurons in this preparation receive input from CPn and/or Tn. CPn input was observed more frequently and the observed postsynaptic currents are predominantly excitatory.

### Input From Ankle Afferents to Shox2 Neurons Involved in Rhythm Generation and Pattern Formation Is Heterogenous but Primarily Inhibitory in the Hemisect Preparation

**FIGURE 3:**
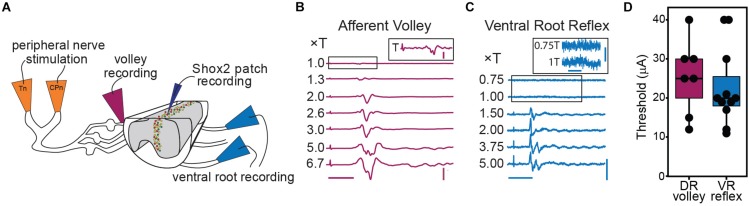
Graded afferent stimulation in the lumbar-hemisected isolated spinal cord preparation. **(A)** Cartoon of hemisected isolated cord preparation from Shox2::cre;Rosa26-lsl-tdTomato;Chx10GFP mice (Shox2^RG^ Shox2^+^Chx10^–^) neurons are red and Shox2^PF^ (Shox2^+^Chx10^+^) neurons are yellow. **(B)** Examples of afferent volley recorded from L4 DR following Tn stimulation. The inset shows the boxed region of the 1.0 × T stimulation on a larger scale for clarity. The deflection on the far left of each trace is the stimulus artifact. Horizontal scale bar is 20 ms. Vertical scale bar is 0.8 mV for main figure and 0.08 mV for inset. **(C)** L4 ventral root reflexes recorded during stimulation of Tn at different stimulation intensities. Threshold was defined as the minimum current necessary to detect a response in the L4 ventral root following CPn or Tn stimulation. The boxed regions of 0.75 and 1.0 × T are shown on a different scale in the inset for clarity. The deflection seen at 1T was small yet consistent. Horizontal scale bars are 20 ms. Vertical scale bar is 0.1 mV for main figure and 0.01 mV for inset. **(D)** Stimulation intensities at threshold for afferent volley and ventral root reflexes following Tn stimulation are plotted. In most preparations, either afferent volley or ventral root reflexes were recorded, but not both.

CPG circuits consist of neuronal elements spread across several lamina and many of these elements are likely removed in the ventral horn-removed preparation (Kiehn and Butt, 2003). However, each lateral half of the lumbar cord is thought to contain a CPG circuit for the ipsilateral hindlimb (Whelan et al., 2000; Frigon et al., 2013; Rybak et al., 2015). In order to preserve ventral horn CPG elements and to record motor activity via the ventral roots, we switched to a hemisect preparation, again in mice ≤ P7 (Figure 3A). In this preparation, afferent volleys and ventral root reflexes (VRR) can be observed following peripheral nerve stimulation (Figures 3B,C). VRR threshold was defined as the lowest stimulation amplitude in which a deflection in the root recording was consistently observed. As the deflection at threshold is very small and comparatively long-lasting, it may correspond to a subthreshold depolarization of the motor neuron pool. As stimulus intensity is increased, we observe a progressive increase in afferent fiber and motor neuron recruitment. In a subset of preparations, we recorded either the afferent volley or VRR following Tn stimulation and quantified the threshold. Afferent volley threshold (25.29 ± 9.52 μA, range 12–40 μA) was not significantly different from VRR threshold (22.64 ± 9.90 μA, range 11–40 μA) but it should be noted that the volley and reflex were not recorded in the same preparation in most cases (Figure 3D). In 3 preparations, both afferent volley and VRR were measured from the same preparation. In these animals, the afferent volley threshold is lower than the VRR threshold.

**FIGURE 4:**
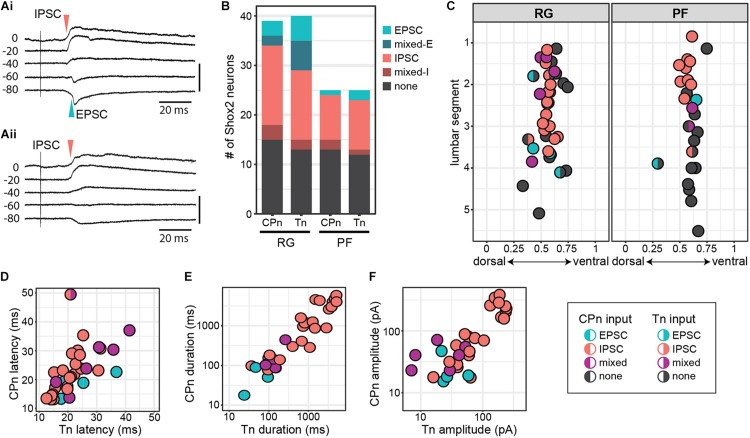
Shox2 neurons which respond to CPn and Tn stimulation respond similarly to both. **(A)** Example traces showing intracellular currents measured from two Shox2 neurons at various membrane holding potentials following Tn stimulation at 2 × T. **(Ai)** A mixed-E response showing inhibitory outward currents (red arrow, upward deflection) followed by excitatory inward currents (green arrow, downward deflection). **(Aii)** A response showing only inhibitory currents with a reversal potential near –60 mV. **(B)** Postsynaptic current types detected in Shox2^RG^ and Shox2^PF^ neurons following stimulation of CPn and Tn. **(C)** Shox2^RG^ and Shox2^PF^ neurons at multiple segmental levels receive postsynaptic currents following CPn and Tn stimulation. Shox2 neurons responding to CPn were significantly more likely to respond to Tn as well (χ^2^ = 31.99, df = 1, *p* = 1.55e-08). Dual responding Shox2 neurons showed a significant association between responses (EPSC, IPSC, mixed) following CPn and Tn stimulation (χ^2^ = 60.06, df = 4, *p* = 2.82e-12), with IPSCs being most common. Data shown as a plot with neuronal position. **(D–F)** In neurons which received postsynaptic currents following CPn and Tn stimulation, response latency, duration and amplitude were positively correlated between CPn and Tn response (linear regression, latency: adjusted *R*^2^ = 0.32, slope = 0.50, *t* = 4.00, *p* = 0.0004; duration: adjusted *R*^2^ = 0.77, slope = 0.83, *t* = 10.35, *p* = 1.41e-11; amplitude: adjusted *R*^2^ = 0.68, slope = 0.61, *t* = 8.35, *p* = 1.95e-09). Duration and amplitude plotted on log scale for clarity.

To target low threshold sensory afferents and avoid activating nociceptive C-fibers, stimulation was delivered at 2 times threshold (×T) for a VRR (Kiehn et al., 1992; Talpalar et al., 2011; Bui et al., 2013). Threshold for VRR was similar between CPn (21 ± 10.2 μA, range 9–45 μA) and Tn (21.9 ± 11.8 μA, range 10–43 μA) (Wilcoxon rank sum test, *W* = 99.5, *p* = 0.96, data not shown). Postsynaptic currents were measured from Shox2 neurons and included in analysis if a postsynaptic current was observed in response to nerve stimulation in at least 80% of trials. The majority (85.5%, 65/76 from 17 mice) of evoked currents occurred in 100% of experimental trials. Most neurons showed responses that were multicomponent. Shox2 neurons were recorded in voltage clamp configuration at membrane potentials from −80 to 10 mV to better isolate excitatory and inhibitory components (Figure 4A). As before, responses were categorized into EPSC, IPSC, mixed-E and mixed-I categories. For most cases of mixed responses, the excitatory and inhibitory components came in a fixed order in response to each stimulus.

In order to more specifically target Shox2 neurons proposed to be involved in rhythm generation and premotor neurons involved in pattern formation, experiments in the hemisect preparation were performed in Shox2cre;R26-lsl-tdTomato;Chx10GFP mice so that recorded neurons could be classified as putative Shox2^RG^ (tomato^+^GFP^–^) or Shox2^PF^ (tomato^+^GFP^+^) based on fluorescent protein expression. 61% (24/39 from 17 mice) of Shox2^RG^ and 48.0% (12/25 from 10 mice) of Shox2^PF^ neurons responded to CPn stimulation (Figure 4B). 67.5% (27/40) of Shox2^RG^ and 52.0% (13/25) of Shox2^PF^ neurons responded to Tn stimulation. No significant difference was detected between the proportion of Shox2^RG^ and Shox2^PF^ neurons which responded to CPn (χ^2^ = 0.65, df = 1, *p* = 0.42) or Tn stimulation (χ^2^ = 0.98, df = 1, *p* = 0.32). In the hemisect preparation, unlike the ventral horn-removed preparation, the overall proportions of Shox2 neurons responding to CPn and Tn stimulations were similar (CPn: 56.25%, 36/64 neurons; Tn: 61.5%, 40/65; χ^2^ = 0.19, df = 1, *p* = 0.67).

Unlike the data from the ventral horn-removed preparations, most recorded responses were inhibitory (Figure 4B). Of the 76 postsynaptic currents measured in Shox2^RG^ and Shox2^PF^ neurons following peripheral nerve stimulation, 14.5% (11/76) were EPSCs, 10.5% (8/76) were mixed-E, 64.5% (49/76) were IPSCs and 10.5% (8/76) were mixed-I. After grouping responses based on Shox2^RG^/Shox2^PF^ and stimulation site, no significant differences were detected in the proportion of response types received (χ^2^ = 9.01, df = 9, *p*-value = 0.44). In some neurons, stimulation at 5 × T was also tested. In these neurons, the earliest current components typically did not shift from inhibitory to excitatory or vice-versa (data not shown).

### Shox2 Neurons That Respond to Tn and CPn Respond Similarly to Both

Although it is predicted that CPG neurons receive synaptic input directly or indirectly from sensory afferents, the organization of those inputs onto CPG neurons is less clear. For example, it is unknown whether distinct populations of CPG neurons receive synaptic input from different sensory groups (i.e., flexor/extensor), or whether a subset of CPG neurons integrate input from multiple sensory afferents. In a subset of recorded Shox2 neurons (63/68, 92.6%), we were able to test evoked responses to both CPn and Tn stimulation. We observed postsynaptic currents in response to both CPn and Tn in 52.4% (33/63) of neurons (dual responders), to CPn only in 3.2% (2/63) of neurons, to Tn only in 9.5% (6/63) of neurons and to neither in 34.9% (22/63) of neurons. Shox2 neurons responding to CPn were significantly more likely to respond to Tn as well (χ^2^ = 31.99, df = 1, *p* = 1.55e-08; Figure 4C).

Interestingly, 97.0% (32/33) of Shox2 dual responders displayed the same type of postsynaptic current (EPSC, IPSC, mixed) following stimulation of either peripheral nerve and this association was statistically significant (χ^2^ = 60.06, df = 4, *p* = 2.82e-12). This concordance was present in both Shox2^RG^ neurons and Shox2^PF^ neurons. Rostral (above the L3 segment) Shox2^PF^ neurons were more likely to receive CPn or Tn input than caudal (L3 segment and below) Shox2^PF^ neurons (χ^2^ = 5.03, df = 1, *p* = 0.025). In contrast, Shox2^RG^ neurons receiving CPn and Tn input did not appear to show a rostrocaudal bias (χ^2^ = 0.41, df = 1, *p* = 0.52). In addition to response type, we also observed a positive correlation between CPn and Tn latency (23.07 ± 7.90 ms, range 12.57–49.89 ms), duration (1321.89 ± 1687.49 ms, range 15.13–5853.43 ms) and amplitude (85.68 ± 87.65 pA, range 7.09–383.21 pA) in Shox2 dual responders (linear regression, latency: adjusted *R*^2^ = 0.32, slope = 0.50, *t* = 4.00, *p* = 0.0004; duration: adjusted *R*^2^ = 0.77, slope = 0.83, *t* = 10.35, *p* = 1.41e-11; amplitude: adjusted *R*^2^ = 0.68, slope = 0.61, *t* = 8.35, *p* = 1.95e-09; Figures 4D–F). Considered together, these results suggest that Shox2 neurons integrate sensory information from flexor-related and extensor-related ankle afferents.

### Input From Ankle Afferents to Shox2 Neurons Is Also Minimally Disynaptic in the Hemisect Preparation

**FIGURE 5:**
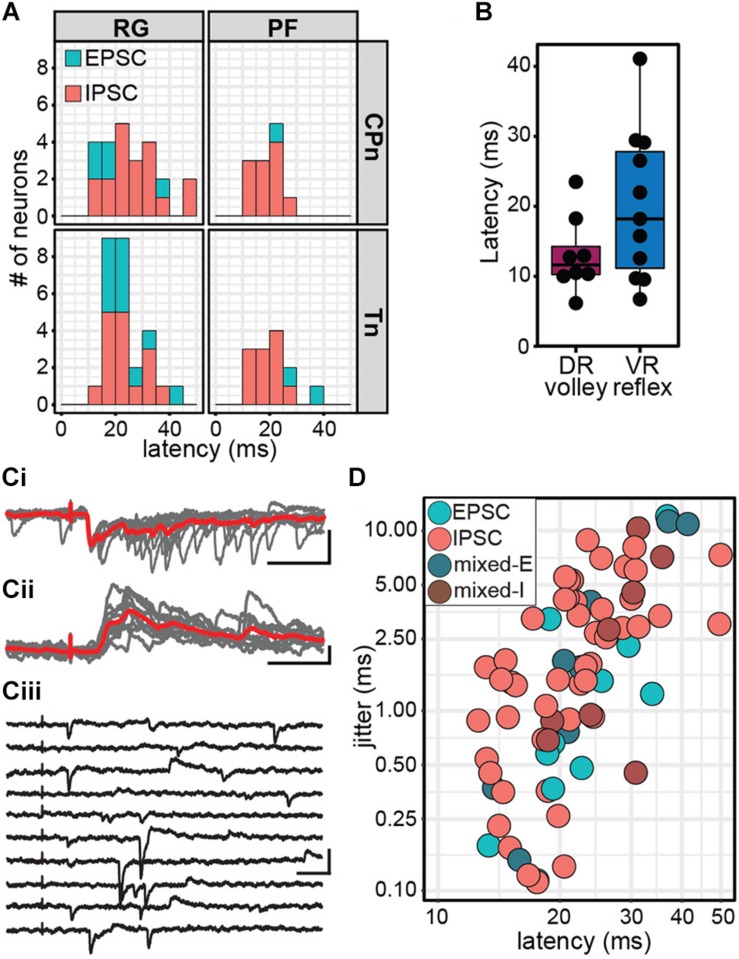
Stimulation of ankle afferents induce postsynaptic currents in Shox2 neurons via a minimally disynaptic pathway in the hemisect preparation. **(A)** Latency distribution of early postsynaptic currents in Shox2^RG^ and Shox2^PF^ neurons following CPn and Tn stimulation. Responses are coded according to polarity of the earliest response component. Inhibitory responses occurred at approximately the same latency as the earliest excitatory responses measured. **(B)** Latencies for dorsal root (DR) volley and ventral root (VR) reflexes were similarly varied. Ventral root reflex latencies spanned nearly the entire range of latencies of evoked currents in Shox2 neurons. **(C)** Example traces showing different Shox2 neurons at different latencies and jitter. Vertical scale bars are 20 pA and horizontal are 50 ms. **(Ci)** Low latency, low jitter excitatory response. Gray traces are 10 individual trials superimposed and red trace is average. **(Cii)** Medium latency, medium jitter inhibitory response. **(Ciii)** High latency, high jitter response showing occasional failures of the earliest component. Jitter is calculated from all sweeps in which any response is seen, resulting in large measured jitter values. **(D)** Latency and jitter of the earliest component of postsynaptic currents observed following CPn and Tn stimulation, color-coded by response subtype. Points plotted on log scale for clarity. A positive relationship was seen between latency and jitter (linear regression, adjusted *R*^2^ = 0.41, *t* = 7.24, *p* = 3.56e-10).

Because response latencies in Shox2 neurons were variable in all preparations used, we conducted a more thorough analysis of response latency in the hemisect preparation to better understand the organization of CPG neuron sensory modulation. Response latencies were highly variable (12.5–49.9 ms) and no difference in latency distribution was detected when the data was separated by whether the earliest response was excitatory or inhibitory (Kolmogorov–Smirnov test, *D* = 0.14, *p* = 0.93; Figure 5A). The earliest latencies of both excitatory (23.72 ± 8.22 ms, range 13.34–41.36 ms) and inhibitory (22.85 ± 7.85 ms, range 12.57–49.89 ms) postsynaptic currents were similar, suggesting that the connections from CPn and Tn nerves to Shox2 neurons are not monosynaptic. No difference in distribution of postsynaptic current latencies was detected between Shox2^RG^ (24.2 ± 8.59 ms, range 13.13–49.89 ms) and Shox2^PF^ neurons (20.73 ± 5.73 ms, range 12.57–36.83 ms) (Kolmogorov–Smirnov test, *D* = 0.23, *p* = 0.25).

For comparison, we also measured the latency of afferent volleys measured at the L4 entry zone and latency of the L4 VRR relative to the onset of stimulation (Figure 5B). As with the measurement of stimulus threshold, it should be noted that the volley and reflex were not recorded in the same preparation in most cases. Because afferent volley latencies (13.04 ± 5.44 ms, range 6.14–23.49 ms) and VRR latencies (20.05 ± 10.64 ms, range 6.70–41.10 ms) were comparable to postsynaptic current latencies, it is difficult to be certain that CPn and Tn input to Shox2 neurons is not monosynaptic based on latency alone. Response jitter has been used as an additional criterion to identify monosynaptic postsynaptic events (Bui et al., 2013; Pujala et al., 2016). We therefore measured the standard deviation of postsynaptic current latency in 10 stimulation trials for each neuron (Figures 5Ci,ii). In some neurons, the earliest postsynaptic components were not reliable, thus resulting in higher reported jitter value (Figure 5Ciii). Unsurprisingly, a positive relationship was seen between latency and jitter (linear regression, slope = 0.24, *t* = 7.24, df = 74, *p* = 3.56e-10; Figure 5D). Jitter for inhibitory responses was 2.65 ± 2.31 ms (range 0.11–8.86 ms); excitatory responses, 2.06 ± 3.44 ms (range 0.11–12.02 ms); mixed-E responses, 3.89 ± 4.60 ms (range 0.15–11.27 ms); and mixed-I responses, 3.47 ± 3.61 ms (range 0.45–10.29 ms). The lower range of jitter for excitatory responses is similar to the lower range of jitter for inhibitory responses, further suggesting that afferent-evoked excitatory currents measured in Shox2 neurons are not monosynaptic. As latency post-stimulation may also be affected by differences in preparations due to age, myelination, conduction pathway length and fluctuations in room temperature, data were also analyzed after normalizing latency by subtracting VRR latency; however, this did not substantially change the results (data not shown). We therefore suggest that both excitatory and inhibitory currents are consistent with a minimally disynaptic pathway from CPn and Tn afferents to Shox2 neurons.

### Response Duration and Amplitude Are Greater in Inhibitory Responses Than Excitatory Responses in the Hemisect Preparation

**FIGURE 6:**
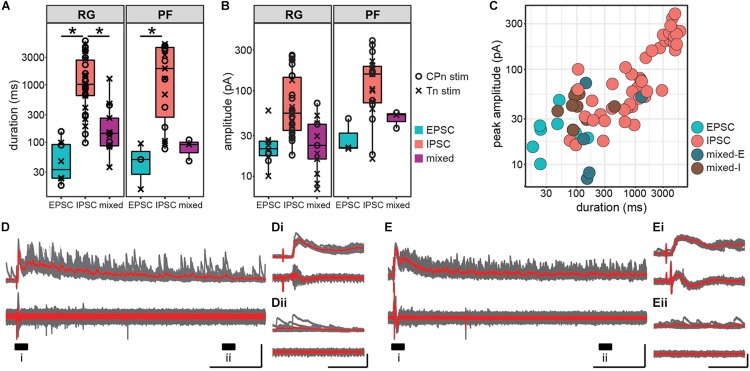
Inhibitory postsynaptic currents in response to ankle afferent stimulation are larger and persist for longer than excitatory postsynaptic currents. **(A)** Duration of postsynaptic currents measured in the hemisect preparation, plotted on log scale for clarity. Current duration was greater for inhibitory responses than for excitatory responses in both the Shox2^RG^ population and the Shox2^PF^ population (*p* < 0.05). **(B)** Absolute peak amplitude of postsynaptic currents, plotted on log scale for clarity. Amplitude for excitatory current components was measured near the inhibitory reversal potential (–50 to –65 mV) and for inhibitory current components near the excitatory reversal potential (0 mV). **(C)** Peak amplitude and duration of postsynaptic currents in individual Shox2 neurons plotted on a log-log scale. Peak amplitude and response duration are positively correlated (linear regression, adjusted *R*^2^ = 0.81, slope = 0.047, *t* = 17.84, df = 74, *p* = 1.60e-28). **(D,E)** Example traces showing large, long duration inhibitory response to afferent stimulation. Shox2 neuron recording is at the top and ventral root recording at the bottom. Ten individual trials (gray) are overlaid with the average of the trials in red. Labeled regions (i, ii) are expanded in insets **(Di,Dii,Ei,Eii)**. In these neurons a low-jitter initial response is followed by a prolonged period in which inhibitory currents continue to be received by Shox2 neurons. Vertical scale bars are 200 pA for intracellular recordings and 0.1 mV for extracellular root recordings. Horizontal scale bars are 1000 ms in **(D,E)** and 100 ms in insets.

In addition to latency and jitter of the early response, we also characterized the duration and peak amplitude of the entire response. Response duration was significantly higher in Shox2 neurons receiving inhibitory inputs (1962.44 ± 1798.97 ms, range 76.16–5853.43 ms) than in Shox2 neurons receiving excitatory (57.75 ± 44.98 ms, range 15.13–153.49 ms) or mixed input (229.30 ± 308.28 ms, range 36.60–1282.00 ms) (Kruskal–Wallis rank sum test, χ^2^ = 38.7, df = 2, *p* = 3.97e-9; *post hoc* Dunn’s test, EPSC-IPSC: *p* = 1.82e-7, EPSC-Mixed: *p* = 0.116, IPSC-Mixed: *p* = 6.99e-5). Differences in duration were also detected when Shox2^RG^ and Shox2^PF^ populations were analyzed separately (Figure 6A). In order to better isolate inhibitory and excitatory current components in pure and mixed responses, amplitudes were respectively measured near the excitatory and inhibitory reversal potentials and reported here as absolute values. Inhibitory amplitudes were 116.32 ± 95.63 pA (range 16.00–383.21 pA), excitatory amplitudes were 26.07 ± 14.33 pA (range 10.11–58.79 pA) and mixed amplitudes were 32.82 ± 18.92 pA (range 7.09–71.44 pA) When comparing Shox2^RG^ and Shox2^PF^ neurons, no significant difference in amplitude was detected for excitatory and mixed currents (excitatory: Wilcoxon rank sum test, *W* = 9, *p* = 0.63; mixed: Welch’s unequal variances *t*-test, *t* = 2.37, df = 5.9, *p* = 0.056). Inhibitory currents observed in Shox2^PF^ neurons (150.0 ± 104.6 pA, range 16.0–383.2 pA) were statistically likely to have greater amplitude than those observed in Shox2^RG^ neurons (95.0 ± 84.4 pA, range 17.6–258.8 pA) (Wilcoxon rank sum test, *W* = 186, *p* = 0.042, *n*1 = 19, *n*2 = 30) (Figure 6B). A positive association was observed between response duration and amplitude (linear regression, adjusted *R*^2^ = 0.81, slope = 0.047, *t* = 17.84, df = 74, *p* = 1.60e-28; Figure 6C). Interestingly, in some neurons we observed sustained current activity, sometimes over 5 s after the initial stimulation. All of these responses were inhibitory and occurred with similar frequency following CPn stimulation and Tn stimulation. In the representative traces shown in Figures 6D,E, an initial large, consistent response can be observed followed by a prolonged period (>1 s) over which many inhibitory currents are seen with variable timing. These results suggest recurrent activation of a circuit with inhibitory synapses on some Shox2 neurons following single pulse stimulation of sensory afferents.

### Both CPn and Tn Stimulation Evoke Flexor-Biased Locomotor Perturbations in P1-3 Mice

It may be surprising that stimulation of CPn and Tn nerves which innervate antagonist muscles both produce similar effects in Shox2 neurons. In many animal models, flexor and extensor afferents generally evoke opposing effects on ongoing locomotion (McCrea, 2001; Rossignol et al., 2006). However, it has been shown that the development of this phenotype does not occur until P4 in the rat and it is possible that a similar developmental switch occurs in the mouse (Iizuka et al., 1997; Hinckley et al., 2010). We therefore applied brief trains of stimulation (50 μs, 20 Hz, 4–6 pulses) during drug-evoked fictive locomotion in whole cord preparations from P1 to P3 mice with attached peripheral nerves (*n* = 7 mice). Stimulation strength was set to 2 × T for VRR for each nerve, measured before locomotor drugs were applied. Ventral root activity was monitored using suction electrodes attached to the flexor-biased L1 VR and the extensor-biased L4 VR. Stimulation was delivered at a regular interval, typically 100 s, which was unrelated to locomotor phase. Therefore, stimulation could occur in any part of the locomotor cycle. Trials where the stimuli occurred during the flexor-related burst period were considered separately from those that occurred during the interburst period. To quantify the effects of CPn and Tn stimulation during ongoing locomotion, we analyzed the cycle period and duty cycle of the flexor-biased ventral root following stimulation either during the flexor-related burst or the interburst period which includes the extensor-related burst. Each cycle was defined as the flexor burst and following interburst period, so stimulation during the burst occurred in the earlier portion of the cycle and stimulation during the interburst period occurred in the later portion of the cycle. As flexor bursts typically occupied less than half of the cycle and stimulations were delivered at 100 s intervals, fewer trials in which stimulation occurred during a burst were available for analysis. Stimulation trials were only considered if the standard deviation of the three cycles preceding stimulation was less than 50% of the mean cycle period. For each animal and condition, cycle periods were averaged and an animal was only considered if the standard deviation of the three averaged cycles preceding stimulation was less than 10% of the mean cycle period.

**FIGURE 7:**
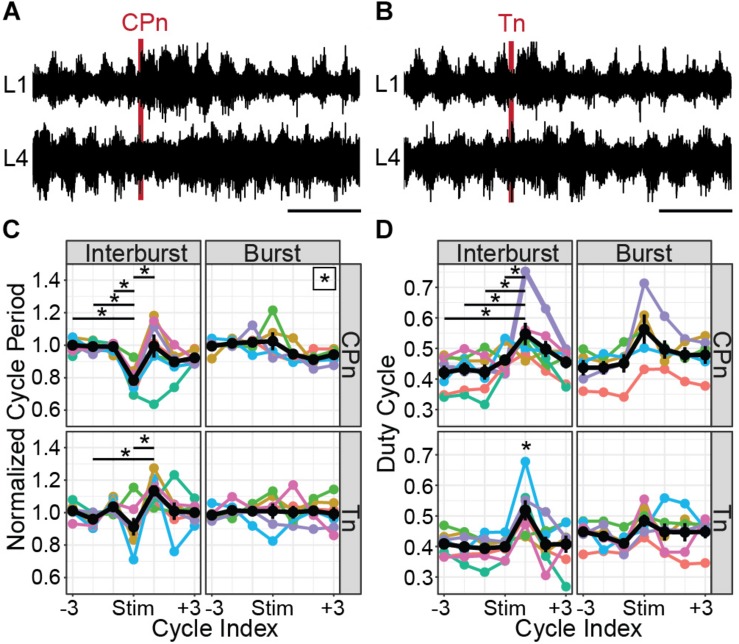
CPn and Tn stimulation trains both induce flexor-biased resetting of drug-evoked fictive locomotion in cords from P1 to P3 mice. **(A)** Representative example of CPn stimulation (50 μs, 20 Hz, 6 pulses) applied during the flexor interburst period of drug-induced locomotion. Top trace shows recording from L1 VR and bottom trace from L4 VR, corresponding to flexor and extensor motor output respectively. Following CPn stimulation (red line), there is truncation of the ongoing extensor burst and initiation of a new flexor burst. **(B)** Representative example of Tn stimulation applied during the flexor interburst period. Following Tn stimulation (red line), there is truncation of the ongoing extensor burst and initiation of a new flexor burst. In some cases, but not always, there is a short delay preceding the flexor burst initiation. **(C)** Normalized cycle period measured from L1 VR recordings before, during and after CPn and Tn stimulation delivered during either the flexor interburst period or burst. Cycles are defined as starting with the flexor burst. Colors correspond to different spinal cord preparations and the black line shows the mean and standard error. When stimulation was delivered during the interburst period, both CPn and Tn stimulation typically reduced the cycle period of the ongoing cycle by truncating the interburst period and increased the cycle period of the subsequent cycle containing the evoked flexor burst. Stars indicate significant pairwise comparison (*p* < 0.05). A star in the upper right corner of the plot indicates significant omnibus test without significant post-hoc comparisons. **(D)** Duty cycle measured from L1 VR recordings before, during and after CPn and Tn stimulation during either the flexor interburst period or burst. Colors correspond to different spinal cord preparations and the black line shows the mean and standard error. Increase in duty cycle for the cycle following stimulation during the interburst period corresponds to an increased burst duration for the evoked flexor burst. A star directly above a point indicates significant pairwise comparisons (*p* < 0.05) with every other point.

Both CPn (Figure 7A) and Tn (Figure 7B) stimulation induced the onset of a new flexor burst and truncated an ongoing extensor burst when applied during the flexor interburst period. Following stimulation, the evoked flexor burst was typically longer than previous flexor bursts and often greater in amplitude. These excitatory effects could sometimes be seen for several cycles. In many cases, a concomitant decrease in amplitude was seen in the extensor bursts following stimulation. This resulted in statistically significant changes in cycle period (Figure 7C). Following CPn stimulation, we observed a reduction of cycle period in the perturbed burst, corresponding to a truncation of the interburst period in 7/7 preparations (Kruskal–Wallis rank sum test, χ^2^ = 26.6, df = 6, *p* = 0.00017). Typically, we also observed an increase of cycle period in the following cycle corresponding to a longer flexor burst. In one preparation, we instead observed a reduction of cycle period in the two cycles following perturbation, corresponding to an increased locomotor frequency. Following Tn stimulation, the evoked flexor burst sometimes appeared immediately and sometimes after a short delay. In both cases, we observed similar reductions in cycle period for the perturbed cycle and increase in cycle period in the cycle after Tn stimulation in 5/7 cords (Kruskal–Wallis rank sum test, χ^2^ = 19.575, df = 6, *p* = 0.0033, *n* = 7 preparations). When stimulation was applied during the burst, significant differences in cycle period were observed following CPn stimulation, but not Tn stimulation (Kruskal–Wallis rank sum test, CPn: χ^2^ = 13.4, df = 6, *p* = 0.037, *n* = 5 preparations; Tn: χ^2^ = 1.65, df = 6, *p* = 0.95, *n* = 6 preparations). However, *post hoc* testing with corrections for multiple comparisons for changes in cycle period following CPn stimulation during the flexor burst did not show any significant pairwise comparisons.

Increases in cycle period can result from prolongation of either the burst or interburst period. We therefore also quantified the duty cycle, defined as the L1 burst duration divided by the cycle period. When stimulation was applied during the interburst period, we again observed significant differences in duty cycle for both CPn and Tn stimulation (Figure 7D). Specifically, there was an increase in the duty cycle for the cycle following stimulation, corresponding to the stimulation-evoked flexor burst that was typically longer in duration [CPn: Kruskal–Wallis rank sum test, χ^2^ = 14.3, df = 6, *p* = 0.027, *n* = 7 preparations; Tn: one-way ANOVA, *F*(6,42) = 3.98, *p* = 0.0030, *n* = 7 preparations]. When stimulation was applied during the flexor burst, no statistically significant change in duty cycle was observed [CPn: one-way ANOVA, *F*(6,28) = 2.05, *p* = 0.091, *n* = 5 preparations; Tn: Kruskal–Wallis rank sum test, χ^2^ = 6.3, df = 6, *p* = 0.39, *n* = 6 preparations].

Taken together, these results demonstrate that flexor and extensor-related ankle afferents both induce flexor-biased resetting of ongoing locomotion in P1–P3 mice. Resetting behavior is more consistent across mice following CPn stimulation than following Tn stimulation and it is possible that this behavior may be different in older mice.

## Discussion

Our experiments were designed to provide insight into the sensory modulation of CPG neurons. We focused on Shox2 neurons, which can be separated into neurons with roles in rhythm generation (Shox2^RG^) and pattern formation (Shox2^PF^). Spinal cord preparations were isolated from neonatal mice and sensory afferents were activated via electrical stimulation of either dorsal root or peripheral nerve while recording from Shox2 neurons. The key findings of our study are that Shox2 neurons with both identities broadly receive postsynaptic currents following afferent stimulation in the quiescent state. In these experiments, Shox2 neurons which responded to activation of flexor and extensor sensory afferents tended to display similar currents to both. Furthermore, we show that in a large subset of medially located Shox2 neurons, the postsynaptic current characteristics following CPn and Tn stimulation were highly associated.

### Sensory Afferent Stimulation Produces Heterogenous Effects on Shox2 Neurons

In all three preparations used in this study, activation of sensory afferents induced postsynaptic currents that were consistent and robust across multiple trials, but which were highly variable between different neurons. We observed EPSCs, IPSCs, mixed responses and non-responders in all preparations with no clear spatial organization and which did not differ according to stimulation site or Shox2^RG^/Shox2^PF^ identity. Recorded postsynaptic currents also occurred with a range of latencies and durations, and with differing numbers of synaptic components.

There are multiple possible explanations for this heterogeneity. Firstly, Shox2 neurons are known to be heterogeneous in many ways. Although deletion experiments have defined locomotor roles for Shox2^RG^ and Shox2^PF^ neurons, it is not clear that every Shox2 neuron is truly part of the CPG. Indeed, although most Shox2 neurons are rhythmically active during pharmacologically evoked fictive locomotion, slightly less than a third of Shox2 neurons are not (Dougherty et al., 2013). Furthermore, rhythmically active Shox2 neurons consist of both flexor- and extensor-aligned pools with no known genetic or electrophysiological marker. Connectivity experiments were performed in the quiescent state and therefore we were unable to identify whether the recorded neurons were flexor-aligned, extensor-aligned or non-rhythmic. Thus, one possible explanation for the mixture of responses seen is that flexor-aligned, extensor-aligned and non-rhythmic Shox2 neurons show stereotypical response patterns which appear heterogeneous in the undifferentiated population.

Another possibility is that the response of CPG neurons to sensory stimulation is strongly modulated by locomotor context. For example, Ib afferents from extensor muscles are known to inhibit extensor motor neurons in the quiescent context via a disynaptic pathway but to activate extensor motor neurons during locomotion (Gossard et al., 1994). Therefore, it is possible that Shox2 neurons may receive functionally different inputs from sensory afferents in a state-dependent manner. Although these studies cannot directly address this possibility, it is known that repetitive sensory stimulation can evoke locomotor-like rhythms, presumably via a pathway synapsing on rhythm-generating circuits. Additionally, activation of CPG circuitry is more reliant on afferent pathways following spinal cord injury (Takeoka et al., 2014; Takeoka and Arber, 2019). Therefore, it is likely that at least some sensory afferents can access rhythm-generating neurons in a functionally relevant manner even in the quiescent state. Specifically, our observation that Shox2 neurons receive afferent input from multiple sensory pathways with differing polarities and latencies in the quiescent state is consistent with the possibility that context-specific inhibitory gating alone could produce powerful and flexible modulation of sensory effects on locomotion.

Finally, the electrical stimulation used in these experiments is relatively non-specific. In particular, dorsal root stimulation will activate many afferent pathways including proprioceptive inputs from flexor, extensor and bifunctional muscles as well as cutaneous afferents. In order to more specifically activate flexor- and extensor-related afferents, we stimulated CPn and Tn respectively. However, CPn and Tn also both contain a mix of proprioceptive and mechanoreceptive afferents. CPn is a hindlimb nerve which innervates muscles and skin of the lateral and anterior compartment of the leg, as well as skin of the foot dorsum and some intrinsic foot muscles. Tn is a hindlimb nerve which innervates the muscles and skin of the posterior compartment of the leg as well as the foot plantar surface and some intrinsic foot muscles. We were primarily interested in understanding proprioceptive input in our experiments and so limited our stimulation to 2 × T for VRR (Kiehn et al., 1992; Talpalar et al., 2011; Bui et al., 2013). However, low strength electrical stimulation will activate fibers from both muscle spindles (Type Ia and II) and Golgi tendon organs (Type Ib), as well as low threshold mechanoreceptors. Thus, it is possible that non-specific activation of different sensory modalities could have evoked the flexor-biased effects that we observed following Tn stimulation. In the cat, enhancement of extensor muscle activity from activation of ankle extensor proprioceptors is Ib dependent and the effect of spindle activation on locomotor phase timing is minimal (Conway et al., 1987). Activation of many cutaneous hindlimb afferents can induce resetting to flexion. However, the foot pad which is innervated by Tn has been shown to have phase-dependent effects during locomotion, enhancing extensors during stance and flexors during swing (Duysens and Pearson, 1976; Duysens, 1977). This phase-dependent pattern is different from the flexor-biased effects we observed, which were strongest during the extensor burst. Therefore, the effects observed in this study are unlikely to result solely from non-specific activation of sensory fiber types.

Taken together, the results suggest that although Shox2 neurons are highly responsive to afferent stimuli, the synaptic pathways interposed between sensory afferents and Shox2 neurons are complex, overlapping and may involve differing numbers and differing populations of interposed neurons.

### Similar Postsynaptic Currents Are Observed Following Both CPn and Tn Stimulation in Shox2 Neurons

Although there was large variability between Shox2 neurons in the effect of sensory afferent stimulation, we nevertheless observed an interesting and consistent pattern in neurons which responded to stimulation of both CPn and Tn. As assessed by presence, polarity, duration and amplitude, postsynaptic currents seen in Shox2 neurons were highly similar following CPn and Tn stimulation in the hemisect preparation. In contrast, many other studies have highlighted the opposing roles of flexor- and extensor-specified afferents on ongoing locomotion (Conway et al., 1987; Guertin et al., 1995; Hiebert et al., 1996; McCrea, 2001; Stecina et al., 2005; Rossignol et al., 2006). As mentioned above, one possible explanation for this discrepancy is that alterations in sensory processing are known to occur during locomotion in a state- and phase-dependent manner and Shox2 behavior was assayed in the quiescent state for these studies. Thus it is possible that if these experiments were repeated in a locomotor context, differential modulation of CPn- and Tn-related postsynaptic currents in Shox2 neurons may result in response patterns more closely aligned with flexor-extensor antagonism. These context-specific changes could result from either central modulation of CPG interneurons or through direct modulation of afferent pathways through mechanisms such as primary afferent depolarization (Forssberg et al., 1975; Andersson et al., 1978; García-Ramírez et al., 2014; Goulding et al., 2014). Another possible explanation for this discrepancy is that these experiments utilized neonatal mice, while the effects of proprioceptive input have been best characterized in the adult animal models. Indeed, in the rat, a developmental switch has been shown to occur at around P4 (Iizuka et al., 1997). Prior to this switch, low-intensity quadriceps nerve stimulation results in flexor burst prolongation whereas during P4–P6 the same stimulation induces flexor burst truncation. Sensory perturbation of locomotion in the mouse model is less well-characterized, but flexor-biased resetting phenotypes have also been reported following stimulation of the predominantly extensor-related L5 dorsal root in young mice (Hinckley et al., 2010). Consistent with this, we demonstrate that trains of either CPn and Tn stimulation both result in a flexor-biased resetting pattern in P1-P3 mice. Although the majority of data collected from the hemisect preparations with peripheral nerve dissections were from mice <P4, the data includes 5 neurons from a P7 mouse, of which 3 were Shox2^RG^ and 2 were Shox2^PF^. Among these neurons, all responded to both CPn and Tn similarly (4 inhibitory and 1 mixed). This may be due to later maturation of sensory locomotor modulation in mice versus rat, differential modulation of sensory pathways between the quiescent and locomotor context (Rossignol et al., 2006), or simply persistence of neonatal connectivity patterns in some neurons at P7 that would be expected to gradually switch with age. Additionally, electrical coupling between Shox2 neurons is prevalent at least to P17 (Ha and Dougherty, 2018). It is possible that gap junctional coupling is leading to the detection of indirect inputs that would not be observed if tested following the decline of electrical connections (Marder et al., 2017). Further exploration of developmental changes in sensory pathways synapsing on CPG elements could provide important insights into locomotor learning and circuit plasticity.

In the ventral horn-removed preparations, these results were less robust but similar. Of the 4 Shox2 neurons in the ventral horn-removed peripheral nerve preparation which responded to both CPn and Tn stimulation, 3 Shox2 neurons showed the same response polarity following both stimulation types and the fourth neuron received excitatory current with CPn stimulation and mixed-E currents with Tn stimulation. It is not clear why the Tn response rate was relatively lower in the ventral horn-removed peripheral nerve preparation than the hemisect preparation, but may suggest a difference specific to the medial-most population accessible for patch clamp in the hemisect preparation or a difference in dorsal-ventral distribution of neurons interposed in the CPn and Tn processing pathways.

Similarly, in the ventral horn-removed dorsal root preparation, a small number of neurons responded to both L2 and L5 stimulation, none of which displayed the fully opposing EPSC-IPSC phenotype although many unsurprisingly displayed mixed currents. Postsynaptic currents in Shox2 neurons were more likely to be observed following L5 than L2 stimulation. Both hip afferents and ankle afferents should have strong access to the rhythm-generating circuitry as both have been shown to be able to modulate step timing and entrain ongoing locomotion (Duysens and Pearson, 1980; Conway et al., 1987; Gossard et al., 1994; Guertin et al., 1995; Perreault et al., 1995; Lam and Pearson, 2001; McVea et al., 2005). Therefore, these results may possibly reflect a differential role for Shox2 neurons in the integration of information from hip and ankle afferents. Shox2^RG^ neurons are known to comprise only a subset of the locomotor rhythm-generating kernel, as deletion of all Shox2 neurons results in slowed locomotion but does not fully eliminate it. Therefore, another neuronal population in the CPG may more strongly integrate information from hip-related afferents.

**FIGURE 8:**
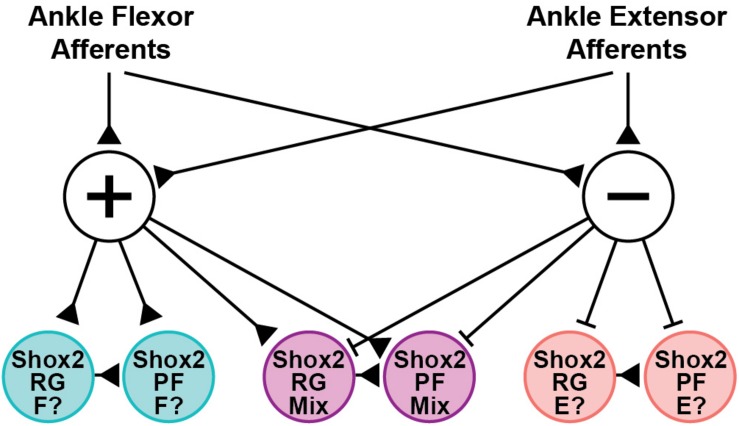
Hypothesized structure of afferent pathways synapsing on Shox2 neurons in the neonate. Ankle flexor-related afferents from the CPn and extensor-related afferents from the Tn synapse on both excitatory (+) and inhibitory (−) interneuron populations. The interposed excitatory interneurons subsequently synapse on three sets of Shox2^RG^ and Shox2^PF^ populations that receive excitatory, inhibitory or mixed inputs. Pathways are depicted disynaptically but may be oligosynaptic. This connectivity structure is proposed on the basis of data collected in the quiescent context, but is consistent with the observed flexor-biased modulation of ongoing locomotion at P1-P3 by activation of CPn and Tn afferents. Together, this suggests that the Shox2 population receiving excitatory inputs (teal) may be flexor-related (F?), while the Shox2 population receiving inhibitory inputs (pink) may be extensor-related (E?). Developmental changes in connectivity either by pruning or by context-dependent activation of gating circuits are expected to occur at the level of the interposed interneurons. Production of connectivity structures in which flexor- and extensor-related Shox2 neurons receive excitatory inputs from homonymous afferents and inhibitory inputs from antagonist afferents, as suggested by studies in the rat and cat, can be more easily explained by pruning or gating if the interposed neurons targeted by CPn and Tn afferents are distinct from one another (not depicted).

Shox2 neurons are proposed to contribute to both the RG and PF layers of the CPG (Dougherty et al., 2013; Rybak et al., 2015). In the hemisect preparation, we identified Chx10-negative Shox2^RG^ and Chx10-positive Shox2^PF^ neurons. Both Shox2^RG^ and Shox2^PF^ neurons responded at similar rates and showed similar patterns of response characteristics. This is consistent with previous experimental data and modeling studies which have predicted that afferent feedback should have access to neurons serving both RG and PF roles (Grillner, 1981; Burke et al., 2001; Rossignol et al., 2006; McCrea and Rybak, 2007). Additionally, both Shox2^RG^ and Shox2^PF^ neurons are known to contribute to flexor- and extensor-aligned populations during locomotion. Therefore, the Shox2 neurons recorded in these experiments should represent a heterogeneous population with some neurons being flexor-aligned and others extensor-aligned. Because both CPn and Tn stimulation during locomotion induced flexor-biased resetting, we would therefore expect neurons that are inhibited to be extensor-aligned and neurons which are excited to be flexor-aligned so long as excitatory/inhibitory response patterns in the quiescent state are predictive of postsynaptic currents in the locomotor state (Figure 8). Flexor-biased Shox2 neurons have previously been reported to comprise the majority of Shox2 neurons, which corresponds with the primarily excitatory currents seen in the ventral horn removed dorsal root and peripheral nerve preparations. However, in the hemisect preparation, most currents are inhibitory. The reasons for this are unclear, but differences in afferent connectivity between the medially located subpopulation of Shox2 neurons that are accessible in the hemisect preparation and the mediolaterally dispersed neurons accessible in the ventral horn-removed preparations may suggest mediolateral patterning of Shox2 neuron function and identity. In this study, we were unable to detect mediolateral connectivity differences within data collected using the ventral horn-removed preparations; however, in a previous study using retrograde viral tracing, the distribution of Shox2 neurons which synapsed on ankle extensor motor neurons extended further medially in comparison to Shox2 neurons which synapsed on ankle flexor motor neurons (Dougherty et al., 2013).

### Long-Lasting Inhibitory Responses Were Observed in a Subset of Shox2 Neurons Recorded From the Hemisect Preparation

In a subset of Shox2 neurons in the hemisect preparation, we also identified a long-lasting inhibitory circuit that synapses on these neurons. This subpopulation is comprised of both Shox2^RG^ neurons and Shox2^PF^ neurons. In these neurons, postsynaptic currents were recorded with durations of up to several seconds, suggesting activation of a recurrently activated circuit which can provided sustained inhibitory currents to Shox2 neurons. It is unclear why these currents were detected only in the hemisect preparation, but two explanations seem plausible. Firstly, the neurons that are accessible in the hemisect preparation are very medial and so this circuit may be spatially organized such that medial neurons are more likely to be activated. Secondly, it is possible that this recurrent circuit is located at least partially in the ventral portion of the cord and therefore is damaged or removed in the ventral horn-removed dorsal root and peripheral nerve preparations. Long-lasting inhibitory effects following peripheral nerve stimulation have previously been reported (Rudneva and Slivko, 2000). It is possible that similar mechanisms and common neuronal substrates are involved in responses observed here. As prolonged trains of dorsal root stimulation can induce locomotion *in vitro*, it is possible that one role for these long-lasting inhibitory currents in response to single pulse stimulation is to prevent inadvertent inappropriate activation of locomotor circuits. Furthermore, as such currents are detected in only a subpopulation of Shox2^RG^ and Shox2^PF^ neurons, it is possible that they may serve to bias the initial CPG state, thus priming the CPG circuitry to produce initial motor activity that is appropriate to the current hindlimb position and load. Another possibility is that these currents may act to modulate circuit excitability. Descending and central modulation of multiple CPG circuit elements and sensory pathways in state and phase-dependent manners are known to be important for the appropriate control of locomotion, and proprioceptive inputs may act similarly (Rossignol et al., 2006; Humphreys and Whelan, 2012; Kim et al., 2017). Modeling experiments have further suggested that neuronal activation level is a critical parameter in the generation of rhythmic activity and that intrinsically rhythmic populations may undergo qualitative shifts from non-active to bursting and then to tonic activity (Shevtsova et al., 2015; Ausborn et al., 2018). Thus, prolonged inhibitory currents may serve to modulate excitatory state during either the initiation or maintenance of locomotor rhythm generation (Nadim et al., 2011). Finally, in computational models of single element oscillators, inhibitory inputs have been shown to have stronger and more reliable effects on phase resetting than do excitatory inputs (Oprisan et al., 2003; Maran et al., 2008). Therefore, these strong inhibitory currents may be important for resetting if they persist in the locomotor state. Based on our data, we are unable to distinguish between which of the above possibilities are more likely, but future experiments will focus on these questions.

## Data Availability Statement

The datasets generated for this study are available on request to the corresponding author.

## Ethics Statement

The animal study was reviewed and approved by the Drexel University Institutional Animal Care and Use Committee.

## Author Contributions

EL and KD conceptualized the study, analyzed the data, and drafted the manuscript. EL, DG-R, and KD designed the experiments. DG-R and EL developed the spinal cord preparations. EL performed the experiments. All authors read, edited, and approved the final manuscript.

## Conflict of Interest

The authors declare that the research was conducted in the absence of any commercial or financial relationships that could be construed as a potential conflict of interest.
